# Multiple Cytokines Are Released When Blood from Patients with Tuberculosis Is Stimulated with *Mycobacterium tuberculosis* Antigens

**DOI:** 10.1371/journal.pone.0026545

**Published:** 2011-11-21

**Authors:** Kathryn L. Kellar, Jennifer Gehrke, Stephen E. Weis, Aida Mahmutovic-Mayhew, Blachy Davila, Margan J. Zajdowicz, Robin Scarborough, Philip A. LoBue, Alfred A. Lardizabal, Charles L. Daley, Randall R. Reves, John Bernardo, Brandon H. Campbell, William C. Whitworth, Gerald H. Mazurek

**Affiliations:** 1 Division of Scientific Resources, Centers for Disease Control and Prevention, Atlanta, Georgia, United States of America; 2 Division of Tuberculosis Elimination, Centers for Disease Control and Prevention, Atlanta, Georgia, United States of America; 3 Department of Medicine, University of North Texas Health Science Center, Fort Worth, Texas, United States of America; 4 Tarrant County Public Health Department, Fort Worth, Texas, United States of America; 5 Oak Ridge Institute for Science and Education, Oak Ridge, Tennessee, United States of America; 6 Naval Hospital Great Lakes, Great Lakes, Illinois, United States of America; 7 The New Jersey Medical School National Tuberculosis Center, University of Medicine and Dentistry of New Jersey, Newark, New Jersey, United States of America; 8 Department of Medicine, University of California San Francisco, San Francisco, California, United States of America; 9 Denver Public Health Department, Denver, Colorado, United States of America; 10 The Pulmonary Center, Boston University School of Medicine, Boston, Massachusetts, United States of America; University of Cape Town, South Africa

## Abstract

**Background:**

*Mycobacterium tuberculosis* (*Mtb*) infection may cause overt disease or remain latent. Interferon gamma release assays (IGRAs) detect *Mtb* infection, both latent infection and infection manifesting as overt disease, by measuring whole-blood interferon gamma (IFN-γ) responses to *Mtb* antigens such as early secreted antigenic target-6 (ESAT-6), culture filtrate protein 10 (CFP-10), and TB7.7. Due to a lack of adequate diagnostic standards for confirming latent *Mtb* infection, IGRA sensitivity for detecting *Mtb* infection has been estimated using patients with culture-confirmed tuberculosis (CCTB) for whom recovery of *Mtb* confirms the infection. In this study, cytokines in addition to IFN-γ were assessed for potential to provide robust measures of *Mtb* infection.

**Methods:**

Cytokine responses to ESAT-6, CFP-10, TB7.7, or combinations of these *Mtb* antigens, for patients with CCTB were compared with responses for subjects at low risk for *Mtb* infection (controls). Three different multiplexed immunoassays were used to measure concentrations of 9 to 20 different cytokines. Responses were calculated by subtracting background cytokine concentrations from cytokine concentrations in plasma from blood stimulated with *Mtb* antigens.

**Results:**

Two assays demonstrated that ESAT-6, CFP-10, ESAT-6+CFP-10, and ESAT-6+CFP-10+TB7.7 stimulated the release of significantly greater amounts of IFN-γ, IL-2, IL-8, MCP-1 and MIP-1β for CCTB patients than for controls. Responses to combination antigens were, or tended to be, greater than responses to individual antigens. A third assay, using whole blood stimulation with ESAT-6+CFP-10+TB7.7, revealed significantly greater IFN-γ, IL-2, IL-6, IL-8, IP-10, MCP-1, MIP-1β, and TNF-α responses among patients compared with controls. One CCTB patient with a falsely negative IFN-γ response had elevated responses with other cytokines.

**Conclusions:**

Multiple cytokines are released when whole blood from patients with CCTB is stimulated with *Mtb* antigens. Measurement of multiple cytokine responses may improve diagnostic sensitivity for *Mtb* infection compared with assessment of IFN-γ alone.

## Introduction

Tuberculosis remains a public health threat and is a leading cause of death worldwide, with an estimated 1.7 million deaths annually [1]. Approximately one third of the world's population are infected with the causative bacterium, *Mycobacterium tuberculosis (Mtb)*, and at risk for developing tuberculosis disease. Without intervention, approximately five to ten percent of those latently infected will develop overt disease and the potential to transmit *Mtb* to others [2].

Measurement of the IFN-γ response to *Mtb* antigens has proven useful in detecting *Mtb* infection, both latent infection and infection manifesting as overt disease [3]–[5]. These assays, referred to as IFN-γ release assays (IGRAs), use enzyme-linked immunosorbent assays (ELISAs) to measure differences in the concentration of IFN-γ, or enzyme-linked immunospot assays (ELISpots) to measure differences in the number of cells that produce IFN-γ, after incubation of whole blood or peripheral blood mononuclear cells (PBMCs) with *Mtb* antigens. Initially, IGRAs assessed response to tuberculin purified protein derivative (PPD) or culture filtrate of *Mtb* [6], [7]. Eventually, proteins that are present in *Mtb* but absent from bacillus Calmette-Guérin (BCG) vaccine strains and most nontuberculous mycobacteria were found to improve IGRA specificity [8]–[12]. Early-secreted antigenic target-6 (ESAT-6), culture filtrate protein 10 (CFP-10), and TB7.7 (also called Rv2654) can induce IFN-γ release, and manufactured peptides representing these *Mtb* proteins are used in various combinations as antigenic stimuli in commercially available IGRAs designed to detect *Mtb* infection [4], [13], [14]. While estimates of IGRA specificity for *Mtb* infection are generally high, estimates of IGRA sensitivity vary widely and in most situations are less than ideal. Estimates of IGRA sensitivity range from 70 to 90% based on pooled data from published reviews involving predominantly adults with *Mtb* infection that was confirmed by culture, i.e., adults with culture-confirmed tuberculosis (CCTB) [3], [4]. Studies in high TB-endemic countries have shown lower IGRA sensitivity than studies in low TB-endemic countries [3]. Suboptimal IGRA sensitivity may result from a reduction in IFN-γ release in patients with active or advanced tuberculosis and the fact that fewer antigenic epitopes are presented by specific *Mtb* antigens compared with PPD. A lack of accurate diagnostic standards hampers attempts to estimate test sensitivity for latent *Mtb* infection. There is no gold diagnostic standard with which to confirm the presence of latent *Mtb* infection. However, recovery of *Mtb* by culture in a patient with symptoms compatible with tuberculosis is adequate confirmation for *Mtb* infection, and has been used in most studies to estimate IGRA sensitivity [3], [4]. Other cytokines used in combination or as alternatives to IFN-γ may improve sensitivity for detecting *Mtb* infection. Use of antigens in combination (e.g., ESAT-6, CFP-10, & TB7.7 combined) may also increase analytic and clinical sensitivity by increasing the number of epitopes available to stimulate lymphocytes, thus increasing the amount of cytokine released.

Numerous cytokines have been implicated in the pathogenesis and control of *Mtb* infection [15]–[26]. Measurement of multiple proinflammatory and anti-inflammatory cytokines, chemokines, and growth factors associated with *Mtb* infection (collectively referred to as cytokines for simplicity) may facilitate its detection.

The objectives of this study were: 1) to assess the responses of multiple cytokines to individual and combinations of *Mtb* antigens, 2) to compare the diagnostic potential of other cytokines with IFN-γ for detecting *Mtb* infection, and 3) to determine if measurement of multiple cytokines can improve detection accuracy. To increase the likelihood of identifying diagnostically important alternative cytokines, we studied subjects categorized with a high degree of confidence into those with *Mtb* infection (i.e., patients with CCTB) and those without infection (low-risk control subjects), and used three different multiplexed assays that examined different panels of cytokines.

## Materials and Methods

### Ethics Statement and Plasma Selection

We previously described comparisons of tuberculin skin test (TST) and QuantiFERON®-TB Gold test (QFT-G, Cellestis Ltd., Victoria, Australia) results among patients with suspected tuberculosis [27] and among low-risk Navy recruits [28]. People were excluded if they were known to have received >7 days of therapy for tuberculosis or latent *Mtb* infection. During these studies, most subjects provided blood for evaluation of additional cytokines and antigens after providing written informed consent as approved by Human Subjects Protection Review Boards at all participating institutions: the Centers for Disease Control and Prevention (CDC) Institutional Review Board (IRB), the Boston University Medical Center IRB, the Colorado Multiple Institutional Review Board for Denver Public Health Department, the University of North Texas Health Science Center at Fort Worth and Texas College of Osteopathic Medicine IRB for the Protection of Human Subjects, the National Naval Medical Center IRB, the University of Medicine and Dentistry of New Jersey IRB, the San Diego State University IRB, and the University of California, San Francisco Committee on Human Research. Subjects consented to provide at least 5 mL of blood for the parent study, but could provide up to 30 mL of blood (to allow evaluation of other tests) if possible without additional venipuncture. When the volume of blood was adequate, one-mL aliquots of heparinized blood were incubated simultaneously with saline (Nil), phytohemagglutinin A (mitogen), and cocktails of overlapping peptides representing *Mtb* antigens, including ESAT-6, CFP-10, TB7.7, and combinations of these antigens (ESAT-6+CFP-10 or ESAT-6+CFP-10+TB7.7), for 16 to 24 hours at 37°C within 12 hours of collection. The peptides simulating ESAT-6 and CFP-10 were identical to those used in QFT-G and the QuantiFERON-TB Gold In-Tube test (QFT-GIT); the peptide representing TB7.7 was identical to that used in QFT-GIT; the peptide cocktails were provided by Cellestis Ltd., Victoria, Australia. After incubation, the plasma supernatants were transferred to micro-tubes in 96-well format, and frozen at −80°C.

**Figure 1:**
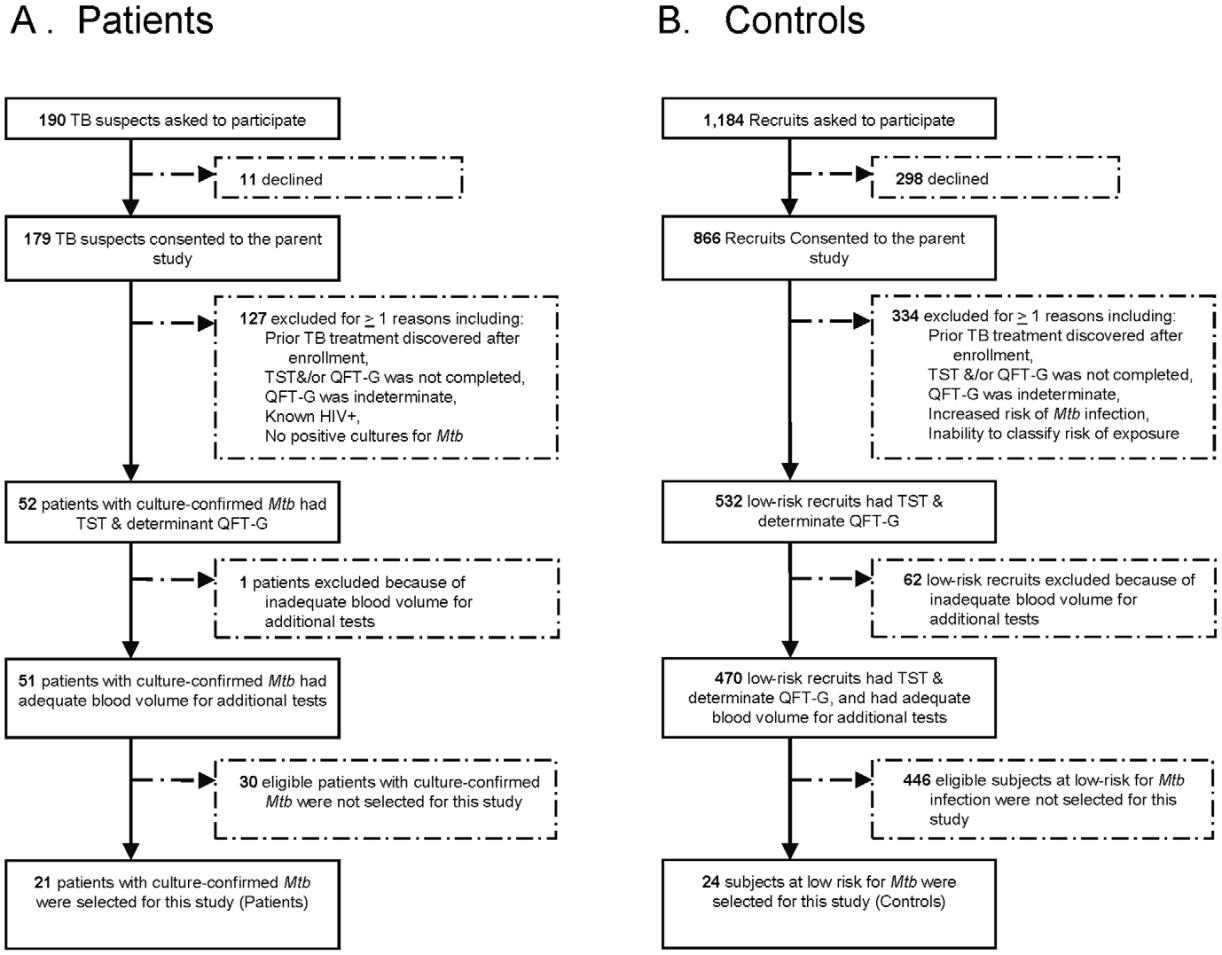
(A–B): Study participation diagram. Panel A depicts selection of 21 patients with culture-confirmed tuberculosis (patients) from among 179 tuberculosis (TB) suspects enrolled in a previously described study [27]. Panel B depicts selection of 24 subjects at low risk for *Mtb* infection (controls) from among 866 Navy recruits enrolled in a previously described study [28]. No significant differences in characteristics of those selected or not selected were observed. Plasma samples from all 21 patients and 24 controls were assessed by the **commercial ELISA**, QuantiFERON-TB Gold test (QFT-G). Plasma samples from 6 patients and 10 controls were assessed by the **in-house MMIA**. Plasma samples from 6 patients and 7 controls were assessed by the **microarray**. Plasma samples from 12 patients and 12 control subjects were assessed by the **commercial MMIA**.

Stored plasma samples from a convenient number of patients (N = 21) with CCTB and from a similar number of subjects (N = 24) at low risk for *Mtb* infection (controls) were selected (as depicted in Figures 1A–B) for evaluation. Funding limitations determined the number of samples assayed. Stored plasma samples from subjects with HIV infection or indeterminate QFT-G results were not included; otherwise, plasma was selected by convenience sampling from lists of patients with CCTB and subjects at low risk of *Mtb* infection without other bias. Clinical and epidemiologic data were collected as part of the parent study. Patients with symptoms or signs of active tuberculosis were considered to have CCTB if *Mtb* was recovered from one or more clinical specimens by culture. Subjects were eligible to be controls and presumed to be uninfected if they denied contact with persons with tuberculosis, were not born (or had not lived >1 month) in a country with an estimated TB prevalence higher than 20 per 100,000 population based on reported nativity and estimates of TB prevalence for 1990 [29], and had not resided, worked or volunteered >1 month at a homeless shelter, prison, drug rehabilitation unit, hospital, or nursing home.

For this study, stored plasma was thawed, aliquoted, and assayed immediately or aliquots were refrozen and thawed once for additional assays. When possible, plasma from the same subject was tested with more than one multiplexed immunoassay. However, this was rare because of limitations in the amount of available plasma and in the allowed number of freeze-thaw cycles.

### Measurement of IFN-γ by ELISA

IFN-γ concentrations were measured by a commercial ELISA using methods and reagents as previously described for the QFT-G [27]. The concentrations of IFN-γ reported in international units/mL (IU/mL) were converted to pg/mL by multiplying by 40 pg/IU [30]. When the optical density for a plasma sample was higher than that of the highest standard, which was common following mitogen stimulation, the value was extrapolated.

### Measurement of Cytokine Concentrations by In-house MMIA

An in-house multiplexed microsphere-based immunoassay (MMIA), also called suspension array, was modified to measure the concentrations of 10 different cytokines. An 8-plex assay that was developed and validated using Luminex technology (Luminex Corp., Austin, TX) for IFN-γ, TNF-α, IL-2, IL-4, IL-6, IL-8, IL-10 and IL-12 (p70) [31], was modified to include VEGF (BioSource/Invitrogen, Carlsbad, CA) and IL-1β (R&D Systems, Minneapolis, MN). The resulting 10-plex cytokine immunoassay was validated by testing for both cross-reactivity of reagents and recovery of spiked multiplexed cytokine standards, as described previously [32], [33]. The assay proceeded as described previously [31]. Briefly, the multiplexed immunoassay was performed by combining all 10 cytokine standards at a concentration of 5000 pg/mL each in phosphate-buffered saline, pH 7.4–0.5% bovine serum albumin −0.02% sodium azide (PBN) and serially diluting the mixture three-fold to obtain concentrations in the range of 5000 to 2.29 pg/mL for the standard curves. Pooled normal human plasma (pretested to ensure undetectable cytokine levels) was added so that the volume of plasma in the standards was equal to the final volume of plasma in each sample. Subject samples were diluted 1∶5 in PBN. The assay proceeded with the sequential addition of a mixture of spectrally distinct fluorescent microspheres (Luminex Corp., Austin, TX), each coupled with a capture antibody for one of the 10 analytes, similarly mixed biotinylated detection antibodies, and finally the reporter, streptavidin phycoerythrin, in a series of incubation steps and washes similar to ELISA methodology. A minimum of one hundred microspheres per cytokine was acquired on a compact flow cytometer (Luminex 100) and the median fluorescent intensity (MFI) of the phycoerythrin conjugate for each microsphere set in each well was reported. Blank values (from wells with all reagents except cytokine or sample) were subtracted from all readings and the concentrations of unknowns were calculated from the standard curves derived with a weighted 5-parameter logistic (5PL) curve-fitting model (Bio-Plex Manager 3.0 and 4.1.1). Values that fell below the lowest range for the standard curve were recorded as 0 pg/mL and upper values were set at 25,000 pg/mL. The precision of the in-house 10-plex assays for this study averaged 7.8% (intra-assay variability, range 5–12% for all cytokines, n = 10). The average lowest range for each cytokine standard was: IL-2 (2.4 pg/mL), IL-4 (2.5 pg/mL), IL-6 (2.7 pg/mL), IL-8 (2.3 pg/mL), IL-10 (2.2 pg/mL), IL-12 (2.4 pg/mL), TNF-α (2.3 pg/mL), IFN-γ (1.9 pg/mL), IL-1β (2.3 pg/mL) and VEGF (7.3 pg/mL) (n = 10, except for VEGF, n = 8; average CV = 0.43).

### Measurement of Cytokine Concentrations by Microarray

A commercial quantitative immuno-microarray (Microarray, Quantibody Human Cytokine Array 1, RayBiotech, Inc., Norcross, GA) was used to measure the concentrations of 20 cytokines in plasma samples; specifically, IFN-γ, IL-1α, IL-1β, IL-2, IL-4, IL-5, IL-6, IL-8, IL-10, IL-12, IL-13, GM-CSF, GRO, MCP-1, MIP-1α, MIP-1β, MMP-9, RANTES, TNF-α, and VEGF were measured. The manufacturer's procedure was followed and the results were analyzed with the RayBiotech Q Analyzer program. Samples and standards were diluted in the Sample Diluent provided with each kit. The standard curves for each cytokine varied in range from a low of 5–400 pg/mL for IL-13 to 370–30,000 pg/mL for MMP-9. The procedure was similar to that used for the in-house MMIA and ELISA, with the detection antibodies labeled with Alexa 555 that was measured with an Axon Gene Pix 4200 A laser scanner (Molecular Devices, Silicon Valley, CA) at a wavelength of 555 and settings of 450, 550 and 650 pmt. Standard curves were analyzed by regression based on linear and logarithmic transformations of the data. The results of any one assay were based on the same pmt readings and data transformation for all cytokines if the correlation coefficient was ≥0.90 for the standard curve. Data not meeting this latter criterion were excluded.

### Measurement of Cytokine Concentrations by a Custom Commercial MMIA

A custom commercial MMIA (Bio-Plex Cytokine Assay, Bio-Rad Laboratories, Hercules, CA) was used to measure 9 cytokines: IFN-γ, IL-2, IL-6, IL-8, IL-12, IP-10, MCP-1, MIP-1β, and TNF-α. This assay was used to examine responses to the combination antigen, ESAT-6+CFP-10+TB7.7, and included samples from additional patients with CCTB and controls. The manufacturer's instructions for performing and interpreting the assay were followed and were similar to those used for the in-house MMIA. Samples and standards were diluted with Human Serum Sample Diluent and Human Serum Standard Diluent, respectively. The ranges of the standard curves varied for each cytokine. When cytokine concentrations were higher than the upper range of the standard curves (i.e. for MCP-1 and MIP-1β), the values were reported as the upper range multiplied by the dilution factor.

### Statistical Analysis

Statistical analyses were conducted with SPSS statistical software (Ver. 15.0, SPSS Inc., Chicago, Ill). The Mann-Whitney U test (also known as the Wilcoxon rank-sum test) was used to compare age of patients with CCTB versus low-risk control subjects. The Chi-squared test or, when an expected frequency was five or less, Fisher's Exact test was used to compare proportions for other subject characteristics. Each cytokine response was determined by subtracting the background cytokine concentration in plasma from blood incubated with saline (Nil) from the cytokine concentration in plasma from blood stimulated with mitogen or *Mtb* antigens. Sensitivity was calculated by dividing the number of patients with CCTB and positive results by the number tested; specificity was calculated by dividing the number of low-risk control subjects with negative results by the number of controls tested. The Mann-Whitney U test was used to compare background cytokine concentrations (and cytokine responses) of patients with CCTB with the background concentrations (or responses) of control subjects. The Wilcoxon signed-rank test was used to compare cytokine responses to different antigens among patients with CCTB. *P* values <0.05 were considered significant. Dot-plots were prepared with Analyse-it for Microsoft Excel v2.20 (Analyse-it Software, Ltd., Leeds, United Kingdom). Histograms were prepared by Microsoft Office Excel 2007.

## Results

Characteristics of the 21 patients with CCTB and the 24 low-risk control subjects selected for this study are summarized in Table 1. Significant differences in age, sex, ethnicity, prevalence of tuberculosis in the country of birth, and BCG vaccination status existed between the two populations. TST induration ranged from 0 to 33 mm (mean = 18, median = 19) among CCTB patients, and was 0 for all control subjects. Because of limitations in the amount of available plasma and in the number of allowed freeze-thaw cycles, none of the plasma specimens were tested in all three multiplexed immunoassays; five patient and 5 control plasmas were analyzed in two different multiplexed assays. These limitations and failure of portions of an assay to meet quality control criteria occasionally caused data to be missing for an antigen or cytokine for a few samples. Characteristics of the 21 CCTB patients tested by the commercial ELISA did not differ significantly from characteristics of the 6 patients tested by in-house MMIA, the 6 patients tested by microarray, or the 12 patients tested by commercial MMIA. Plasmas from a similar number of CCTB patients and control subjects were tested with each assay.

**Table 1:** Demographic and clinical characteristics of participants.

	Patients	Controls	*p-*value
Number of subjects participating in analysis	21	24	
Mean age in years	43	20	
Median age	45	20	<0.001
Minimum age	21	17	
Maximum age	80	25	
Sex: Male	8 (38.1)	23 (95.8)	<0.001
Race/Ethnicity - Number (percentage)			
White non-Hispanic	1 (4.8)	16 (66.7)	<0.001
Black non-Hispanic	5 (23.8)	5 (20.8)	
Asian, Pacific Islander, or Native American	5 (23.8)	1 (4.2)	
Hispanic	10(47.6)	2 (8.3)	
Born in a country with indicated TB burden - Number (percentage)			
<20 cases per 100,000 population	8 (38.1)	24 (100)	<0.001
20 to 100 cases per 100,000 population	5 (23.8)	0 (0)	
>100 cases per 100,000 population	8 (38.1)	0 (0)	
Resided in a country with indicated TB burden (other than birth) - Number (Percentage)			
<20 cases per 100,000 population	18 (85.7)	24 (100)	<0.16
20 to 100 cases per 100,000 population	2 (9.5)	0 (0)	
>100 cases per 100,000 population	1 (4.8)	0 (0)	
Employed or stayed in a health care facility for >1 month- Number (Percentage)	4 (19.0)	0 (0)	0.04
Employed or stayed in a correctional facility for >1 month- Number (Percentage)	5 (23.8)	0 (0)	0.02
History of BCG vaccination- Number (Percentage)			
None	9 (42.9)	24 (100)	<0.001
Unknown	3 (9.5)	0 (0)	
Vaccinated	10 (47.6)	0 (0)	

Characteristics are listed for 21 patients with culture-confirmed tuberculosis (patients) and 24 subjects at low-risk for *M. tuberculosis* (*Mtb*)exposure (controls) who provided samples that were tested in this study. Samples from 5 patients and 6 controls were tested with more than one multiplexed immunoassay. Percentages of the patients or controls in the different categories are indicated in parentheses. The Mann-Whitney U test was used to compare age. Other *p* values were calculated by the Chi-squared test or, when an expected frequency was five or less, a Fisher's Exact test.

As shown in Table 2 for measurements by commercial ELISA, highly significant differences in IFN-γ responses to ESAT-6, CFP-10, TB7.7, ESAT-6+CFP-10, and ESAT-6+CFP-10+TB7.7 were observed for CCTB patients compared with controls (*p* for each comparison <0.001). While the difference in the median IFN-γ response to TB7.7 for patients compared with controls was small (3 vs. 0 pg/mL), the difference was statistically significant (*p*<0.001). As shown in Table 3 for CCTB patients, the difference in IFN-γ response to ESAT-6+CFP-10 versus ESAT-6+CFP-10+TB7.7 was not significant (*p* = 0.327), but responses to the combination antigens were significantly greater than the responses to the individual antigens (*p* ranged from ≤0.001 to 0.007). When a cutoff of 14 pg/mL (equivalent to the 0.35 IU/ml cutoff approved by the FDA for the QFT-G and QuantiFERON-TB Gold In-Tube test) was used to separate positive and negative responses to ESAT-6+CFP-10+TB7.7, nineteen (95%) of 20 CCTB patients were classified as positive and 23 (100%) of 23 controls were classified as negative (one patient and one control did not have ELISA results for plasma stimulated with ESAT-6+CFP-10+TB7.7). None of the *Mtb* antigens, including ESAT-6+CFP-10+TB7.7, induced a measureable IFN-γ response for one patient with CCTB.

**Table 2:** Cytokine concentrations and responses measured with a commercial ELISA.

Cytokine	Subject Group	Nil	Mitogen Response	ESAT-6 Response	CFP-10 Response	TB7.7 Response	ESAT-6+CFP-10 Response	ESAT-6+CFP-10+TB7.7 Response
IFN-γ	Patients	8	524	61	128	3	187*	202*
		(2 to 18)	(28 to 1,035)	(10 to 756)	(0 to 676)	(−2 to 124)	(−1 to 757)	(−2 to 757)
	Controls	4	665	0	0	0	0	0**
		(3 to 15)	(103 to 2,040)	(−7 to 9)	(−8 to 8)	(−8 to 4)	(−4 to 1)	(−7 to 1)
	*p*-value	0.062	0.158	**<0.001**	**<0.001**	**<0.001**	**<0.001**	**<0.001**

Median background IFN-γ concentrations (Nil, pg/mL) and IFN-γ responses (pg/mL) to phytohemagglutinin (Mitogen Response), early secreted antigenic target-6 (ESAT-6 Response), culture filtrate protein 10 (CFP-10 Response), TB7.7 (TB7.7 Response), and combinations of these *M. tuberculosis* (*Mtb*) antigens (ESAT-6+CFP-10 Response, and ESAT-6+CFP-10+TB7.7 Response) are listed with ranges in parentheses. Cytokine concentrations were measured with a commercial enzyme-linked immunosorbent assay (ELISA) for patients with culture-confirmed tuberculosis (patients) and subjects at low-risk of *Mtb* exposure (controls). Responses were calculated by subtracting the background cytokine concentration in plasma from blood incubated with saline (Nil) from the cytokine concentration in plasma from blood incubated with mitogen or *Mtb* antigens. N = 21 for patients and N = 24 for controls unless indicated (*n = 20, **n = 23). *P* values were calculated by Mann-Whitney U Rank Sum test comparing cytokine concentrations or responses for patients with controls. Significant differences (*p*<0.05) are indicated in **bold type**.

**Table 3:** Comparisons of IFN-γ responses to different antigens measured by commercial ELISA among patients with culture-confirmed tuberculosis.

Cytokine	ESAT-6	ESAT-6	ESAT-6	ESAT-6	CFP-10	CFP-10	CFP-10	TB7.7	TB7.7	ESAT-6+CFP-10
	*vs.*	*vs.*	*vs.*	*vs.*	*vs.*	*vs.*	*vs.*	*vs.*	*vs.*	*vs.*
	CFP-10	TB7.7	ESAT-6+CFP-10	ESAT-6+CFP-10+TB7.7	TB7.7	ESAT-6+CFP-10	ESAT-6+CFP-10+TB7.7	ESAT-6+CFP-10	ESAT-6+CFP-10+TB7.7	ESAT-6+CFP-10+TB7.7
IFN-γ	61	61	61	61	128	128	128	3	3	187
	*vs.*	*vs.*	*vs.*	*vs.*	*vs.*	*vs.*	*vs.*	*vs.*	*vs.*	*vs.*
	128	3	187	202	3	187	202	187	202	202
	(0.794)	**(<0.001)**	**(0.007)**	**(0.006)**	**(<0.001)**	**(0.005)**	**(0.005)**	**(<0.001)**	**(<0.001)**	(0.327)

Median IFN-γ responses (from Table 2) to early secreted antigenic target-6 (ESAT-6), culture filtrate protein 10 (CFP-10), TB7.7, and combinations of these antigens for patients with culture-confirmed tuberculosis (patients) are listed with p values in parenthesis calculated by Mann-Whitney U Rank Sum test. Significant differences (*p*<0.05) are indicated in **bold type**.

Cytokine responses measured by in-house MMIA are shown in Table 4. Comparisons of responses to ESAT-6 show significantly greater responses for CCTB patients compared with controls for IFN-γ (94 vs. 0 pg/mL, *p* = 0.006), IL-2 (116 vs. 0 pg/mL, *p*<0.001), and IL-8 (207 vs. 23 pg/mL, *p* = 0.003). Similarly, significant differences in these cytokine responses, as well as IL-6 responses, were found following stimulation with CFP-10, ESAT-6+CFP-10, and ESAT-6+CFP-10+TB7.7. In contrast to findings with ELISA, no difference in IFN-γ response to TB7.7 was observed between patients and controls with the in-house MMIA (*p* = 0.094), which had a much smaller sample size (N = 16 vs. N = 45). Similarly, no differences in responses to TB7.7 were observed between patients and controls with other cytokines included in the in-house MMIA. IL-1β, IL-4, IL-10, and TNF-α responses to antigen stimulation were generally very low. Several control subjects had detectable IL-12 responses but responses to mitogen were low. The background values for VEGF were higher for CCTB patients compared with controls. As shown in Table 5, responses for CCTB patients to combination antigens tended to be greater (lower *p* values) than for individual antigens for IFN-γ, IL-2, IL-6, and IL-8.

**Table 4:** Cytokine concentrations and responses measured with an in-house MMIA.

Cytokine	Subject Group	Nil	Mitogen Response	ESAT-6 Response	CFP-10 Response	TB7.7 Response	ESAT-6+CFP-10 Response	ESAT-6+CFP-10+TB7.7 Response
IFN-γ	Patients	0	4,651	94	349	0	366	380
		(0 to 101)	(229 to 8,890)	(0 to 629)	(109 to 902)	(−4 to 58)	(132 to 590)	(255 to 1,570)
	Controls	8	3,743	0	0	0	0	0
		(0 to 62)	(986 to 21,395)	(−41 to 12)	(−8 to 23)	(−31 to 0)	(−23 to 39)	(−22 to 10)
	*p*-value	0.550	0.828	**0.006**	**0.001**	0.094	**0.001**	**0.001**
IL-1β	Patients	0	19	0	0	0	0	0
		(0 to 16)	(−11 to 53)	(0 to 1)	(0 to 3)	(−4 to 0)	(−3 to 0)	(−2 to 167)
	Controls	0	7	0	0	0	0	0
		(0 to 0)	(0 to 73)	(0 to 0)	(0 to 0)	(0 to 0)	(0 to 0)	(0 to 0)
	*p*-value	0.197	0.821	0.197	0.197	0.197	0.197	0.426
IL-2	Patients	0	281	116	216	0	238	300
		(0 to 14)	(0 to 1,134)	(0 to 293)	(0 to 424)	(0 to 1)	(0 to 532)	(0 to 711)
	Controls	0	543	0	0	0	0	0
		(0 to 0)	(210 to 1,156)	(0 to 0)	(0 to 0)	(0 to 0)	(0 to 12)	(0 to 16)
	*p*-value	0.197	0.329	**0.001**	**0.001**	0.197	**0.002**	**0.006**
IL-4	Patients	0	51	0	0	0	0	0
		(0 to 17)	(0 to 78)	(−17 to 13)	(−17 to 0)	(−17 to 16)	(−17 to 0)	(−17 to 0)
	Controls	12	55	0	0	0	0	0
		(0 to 41)	(−3 to 198)	(−6 to 11)	(0 to 38)	(−14 to 27)	(−41 to 18)	(−9 to 103)
	*p*-value	0.120	0.625	0.663	0.048	0.487	0.152	0.232
IL-6	Patients	11	3,828	15	20	0	20	151
		(0 to 60)	(372 to 23,840)	(0 to 28)	(10 to 55)	(−21 to 9)	(12 to 14,558)	(7 to 22,167)
	Controls	0	3,467	0	0	0	1	0
		(0 to 12)	(2,329 to 13,567)	(−12 to 22)	(−12 to 18)	(−12 to 0)	(0 to 24)	(−12 to 29)
	*p*-value	0.114	1.000	0.066	**0.002**	1.000	**0.015**	**0.005**
IL-8	Patients	91						
	4,539							
	207	1,227	94					
	1,051							
	2,274							
		(0 to 157)	(412 to 7,790)	(117 to 1,842)	(187 to 3,086)	(−2 to 213)	(644 to 24,871)	(336 to 24,814)
	Controls	105	2,151	23	14	37	21	40
		(15 to 290)	(939 to 13,360)	(−106 to 187)	(−40 to 43)	(−43 to 193)	(−30 to 208)	(−44 to 193)
	*p*-value	0.625	1.000	**0.003**	**0.001**	0.278	**0.001**	**0.001**
IL-10	Patients	0	105	0	0	0	0	0
		(0 to 24)	(0 to 653)	(0 to 1)	(−5 to 0)	(−8 to 0)	(−4 to 50)	(0 to 20)
	Controls	0	206	0	0	0	0	0
		(0 to 0)	(60 to 404)	(0 to 13)	(0 to 0)	(0 to 0)	(0 to 0)	(0 to 0)
	*p*-value	0.197	0.448	0.750	0.197	0.197	1.000	0.059
IL-12	Patients	0	0	0	0	0	0	0
		(0 to 20)	(−21 to 0)	(0 to 4)	(−3 to 0)	(−3 to 0)	(−3 to 0)	(−4 to 0)
	Controls	10	−16	1	0	0	1	1
		(0 to 24)	(−48 to 11)	(−8 to 34)	(0 to 26)	(−18 to 22)	(−3 to 19)	(−10 to 23)
	*p*-value	0.169	0.245	0.339	0.112	0.720	0.083	0.223
TNF-α	Patients	0	159	0	0	0	0	1
		(0 to 23)	(0 to 828)	(0 to 6)	(0 to 0)	(−5 to 0)	(−1 to 0)	(0 to 91)
	Controls	0	49	0	0	0	0	0
		(0 to 0)	(21 to 365)	(0 to 0)	(0 to 0)	(0 to 0)	(0 to 0)	(0 to 0)
	*p*-value	0.197	0.329	0.197	1.000	0.197	0.197	**0.017**
VEGF	Patients	737	−1,075	118	−58	133	−104	−153
		(358 to 954)	(−1,538 to −525)	(−128 to 842)	(−242 to 665)	(−178 to 546)	(−291 to 411)	(−680 to 0)
	Controls	144*	−109*	87*	161*	229*	96*	74*
		(0 to 758)	(−1,415 to 0)	(−258 to 279)	(−119 to 208)	(−144 to 519)	(−162 to 519)	(−407 to 443)
	*p*-value	**0.037**	0.109	0.631	0.522	0.631	0.109	0.173

Median background cytokine concentrations (Nil, pg/mL) and cytokine responses (pg/mL) to phytohemagglutinin (Mitogen Response), early secreted antigenic target-6 (ESAT-6 Response), culture filtrate protein 10 (CFP-10 Response), TB7.7 (TB7.7 Response), and combinations of these *M. tuberculosis* (*Mtb*) antigens (ESAT-6+CFP-10 Response and ESAT-6+CFP-10+TB7.7 Response) are listed with ranges in parentheses. Cytokine concentrations were measured with an in-house multiplexed microsphere-based immunoassay (in-house MMIA) for patients with culture-confirmed tuberculosis (patients) and subjects at low-risk of *Mtb* exposure (controls). For concentrations that were designated “out of range” by Bio-Plex Manager software, low values are reported as 0 and high values are reported as the upper limit of the standard curve multiplied by the dilution factor (25,000 pg/mL). Responses were calculated by subtracting the background cytokine concentration in plasma from blood incubated with saline (Nil) from the cytokine concentration in plasma from blood incubated with mitogen or *Mtb* antigens. N = 6 for patients and N = 10 for controls unless indicated (*n = 6). *P* values were calculated by Mann-Whitney U Rank Sum test comparing cytokine concentrations or responses for patients with controls. Significant differences (*p*<0.05) are indicated in **bold type**.

**Table 5:** Comparisons of cytokine responses to different antigens measured by in-house MMIA among patients with culture-confirmed tuberculosis.

Cytokine	ESAT-6	ESAT-6	ESAT-6	ESAT-6	CFP-10	CFP-10	CFP-10	TB7.7	TB7.7	ESAT-6+CFP-10
	*vs.*	*vs.*	*vs.*	*vs.*	*vs.*	*vs.*	*vs.*	*vs.*	*vs.*	*vs.*
	CFP-10	TB7.7	ESAT-6+CFP-10	ESAT-6+CFP-10+TB7.7	TB7.7	ESAT-6+CFP-10	ESAT-6+CFP-10+TB7.7	ESAT-6+CFP-10	ESAT-6+CFP-10+TB7.7	ESAT-6+CFP-10+TB7.7
IFN-γ	94	94	94	94	349	349	349	0	0	366
	*vs.*	*vs*	*vs*	*vs*	*vs*	*vs*	*vs*	*vs*	*vs*	*vs*
	349	0	366	380	0	366	380	366	380	380
	**(0.028)**	**(0.043)**	(0.075)	**(0.028)**	**(0.028)**	(0.463)	(0.116)	**(0.028)**	**(0.028)**	(0.345)
IL-2	116	116	116	116	216	216	216	0	0	0238
	*vs.*	*vs*	*vs*	*vs*	*vs*	*vs*	*vs*	*vs*	*vs*	*vs*
	261	0	238	300	0	238	300	238	300	300
	**(0.043)**	**(0.043)**	(0.138)	**(0.043)**	**(0.043)**	(0.500)	(0.080)	**(0.043)**	**(0.043)**	(0.500)
IL-6	15	15	15	15	20	20	20	0	0	20
	*vs.*	*vs*	*vs*	*vs*	*vs*	*vs*	*vs*	*vs*	*vs*	*vs*
	20	0	20	151	0	20	151	20	151	151
	(0.058)	(0.075)	(0.074)	(0.116)	**(0.028)**	(0.917)	(0.173)	**(0.028)**	**(0.028)**	(0.753)
IL-8	207	207	207	207	1,227	1,227	1,227	94	94	1,051
	*vs.*	*vs*	*vs*	*vs*	*vs*	*vs*	*vs*	*vs*	*vs*	*vs*
	1,227	94	1,051	2,274	94	1,051	2,274	1,051	2,274	2,274
	**(0.028)**	**(0.046)**	(0.249)	**(0.028)**	**(0.028)**	(0.917)	(0.463)	**(0.028)**	**(0.028)**	(0.753)

Median cytokine responses (from Table 4) to early secreted antigenic target-6 (ESAT-6), culture filtrate protein 10 (CFP-10), TB7.7, and combinations of these *M. tuberculosis* (*Mtb*) antigens for patients with culture-confirmed tuberculosis (patients) are listed with p values in parenthesis calculated by Mann-Whitney U Rank Sum test. Significant differences (*p*<0.05) are indicated in **bold type**.

As shown in Tables 6 and 7, microarray analysis for 20 cytokines confirmed that IFN-γ, IL-2, IL-8, and variably IL-6 responses to *Mtb* antigens were significantly greater for patients with CCTB than for controls. In addition, MCP-1 and MIP-1β responses to the *Mtb* antigens tested were significantly higher for patients than controls, and MIP-1α responses were significantly greater for the antigen combinations. No consistent trend in differences between individual and combinations of *Mtb* antigens was evident for patients with this method (Table 8).

**Table 6:** Cytokine concentrations and responses measured with a microarray.

Cytokine	Subject Group	Nil	ESAT-6 Response	CFP-10 Response	ESAT-6+CFP-10 Response	ESAT-6+CFP-10+TB7.7 Response
IFN-γ	Patients	37	682	763	1,270	1,259
		(0 to 1,679)	(87 to 3,600)	(29 to 1,960)	(42 to 5,808)	(−639 to 7,977)
	Controls	286	11	6	8	2
		(8 to 74,712)	(−4,026 to 720)	(−4,738 to 831)	(−15,218 to 642)	(−14,924 to 22)
	*p-*value	0.199	**0.022**	**0.015**	**0.010**	**0.025**
IL-1α	Patients	4	0	0	0	47
		(1 to 413)	(−162 to 14)	(−411 to 56)	(−411 to 207)	(−411 to 95)
	Controls	374	0	0	24	71
		(1 to 2,859)	(−277 to 666)	(−184 to 2,251)	(−358 to 2,107)	(−511 to 306)
	*p-*value	0.152	0.883	0.883	0.775	0.423
IL-1β	Patients	3	0	0	0	30
		(1 to 133)	(−14 to 32)	(−48 to 27)	(−85 to 53)	(−115 to 106)
	Controls	17	0	0	−9	−5
		(0 to 1,101)	(−159 to 218)	(−125 to 333)	(−345 to 33)	(−409 to 27)
	*p-*value	0.520	0.319	0.868	0.289	0.173
IL-2	Patients	0	428	499	740	746
		(0 to 92)	(23 to 3,248)	(39 to 2,251)	(53 to 5,068)	(64 to 6,820)
	Controls	7	2	0	4	0
		(0 to 423)	(−14 to 66)	(−26 to 74)	(−112 to 87)	(−170 to 11)
	*p-*value	0.283	**0.007**	**0.004**	**0.004**	**0.004**
IL-4	Patients	3	0	0	0	0
		(0 to 374)	(−120 to 62)	(−120 to 0)	(−207 to 378)	(−120 to 19)
	Controls	131	0	0	47	35
		(0 to 3,291)	(−136 to 51)	(−110 to 434)	(−591 to 433)	(−1,400 to 291)
	*p-*value	0.430	0.557	0.351	0.304	0.245
IL-5	Patients	1	0	0	0	0
		(0 to 172)	(−53 to 27)	(−115 to 6)	(−133 to 15)	(−168 to 53)
	Controls	6	0	8	0	0
		(0 to 783)	(−18 to 126)	(0 to 166)	(−167 to 146)	(−252 to 24)
	*p-*value	0.519	0.756	0.062	0.769	0.442
IL-6	Patients	7	250	28	182	781
		(0 to 239)	(2 to 874)	(−36 to 378)	(−35 to 1,639)	(−29 to 14,727)
	Controls	32	1	0	2	8
		(1 to 1,914)	(−331 to 21)	(−80 to 151)	(−413 to 143)	(−706 to 26)
	*p-*value	0.174	**0.010**	0.116	0.153	**0.025**
IL-8	Patients	124*	1,038*	1,988*	1,595*	3,076*
		(27 to 595)	(132 to 4,912)	(469 to 4,913)	(933 to 4,913)	(1,002 to 4,911)
	Controls	483**	−11**	75**	−106**	−85*
		(119 to 1,274)	(−174 to 78)	(−61 to 654)	(−376 to 382)	(−303 to 189)
	*p-*value	0.221	**0.014**	**0.027**	**0.014**	**0.021**
IL-10	Patients	2	0	0	0	0
		(0 to 27)	(−19 to 5)	(−27 to 3)	(−21 to 52)	(−27 to 27)
	Controls	55/24	−5/−1	34/6	24/12	−8/−3**
		(1 to 187)	(−20 to 10)	(−3 to 186)	(−3 to 71)	(−56 to 37)
	*p-*value	0.314	0.712	0.097	0.201	0.518
IL-12	Patients	0	0	0	2	2
		(0 to 31)	(−19 to 7)	(−22 to 3)	(−30 to 7)	(−31 to 17)
	Controls	1	0	2	12	1
		(0 to 743)	(−5 to 16)	(0 to 54)	(−18 to 48)	(−45 to 164)
	*p-*value	0.283	0.469	0.151	0.153	0.936

Median background cytokine concentrations (Nil, pg/mL) and cytokine responses (pg/mL) to phytohemagglutinin (Mitogen Response), early secreted antigenic target-6 (ESAT-6 Response), culture filtrate protein 10 (CFP-10 Response), TB7.7 (TB7.7 Response), and combinations of these *M. tuberculosis* (*Mtb*) antigens (ESAT-6+CFP-10 Response and ESAT-6+CFP-10+TB7.7 Response) are listed with ranges in parentheses. Cytokine concentrations were measured by a commercial quantitative immuno-microaray (microarray) for patients with culture-confirmed tuberculosis (patients) and subjects at low risk for *Mtb* exposure (controls). Responses were calculated by subtracting the background cytokine concentration in plasma from blood incubated with saline (Nil) from the cytokine concentration in plasma from blood incubated with mitogen or *Mtb* antigens. Cytokine responses for some patients and controls were not determined due to poor performance of the respective standard curves. N = 5 or 6 for patients subjects and N = 6 or 7 for controls unless indicated (*n = 4, **n = 5). *P* values were calculated by Mann-Whitney U Rank Sum test comparing cytokine concentrations or responses for patients with controls. Significant differences (*p*<0.05) are indicated in **bold type**.

**Table 7:** Cytokine concentrations and responses measured with a microarray.

Cytokine	Subject Group	Nil	ESAT-6 Response	CFP-10 Response	ESAT-6+CFP-10 Response	ESAT-6+CFP-10+TB7.7 Response
IL-13	Patients	0	6	2	7	8
		(0 to 40)	(0 to 32)	(0 to 21)	(0 to 21)	(−5 to 17)
	Controls	12	0	2	0	0
		(0 to 484)	(−33 to 49)	(0 to 172)	(−89 to 184)	(−140 to 3)
	*p-*value	0.115	0.192	0.664	0.174	0.076
GM-CSF	Patients	3	0	4	12	14
		(0 to 112)	(−5 to 44)	(−29 to 32)	(−50 to 107)	(−78 to 115)
	Controls	17	2	0	6	0
		(0 to 540)	(−3 to 108)	(−18 to 65)	(−117 to 22)	(−272 to 23)
	*p-*value	0.390	0.469	0.717	0.431	0.197
GRO	Patients	2,811	735	706	829	880
		(183 to 3,875)	(−1,313 to 2,384)	(−1,083 to 4,907)	(−2,008 to 7,074)	(−2,547 to 37,398)
	Controls	2,468	71	−42	−96	−150
		(387 to 86,052)	(−769 to 1,661)	(−499 to 4,487)	(−14,640 to 2,782)	(−23,190 to 833)
	*p-*value	0.475	0.668	0.668	0.391	0.200
MCP-1	Patients	20,392	72,572	102,845	76,846	72,438
		(5,954 to 276,527)	(26,792 to 273,723)	(39,217 to 273,765)	(56,043 to 273,741)	(34,099 to 273,614)
	Controls	10,289	641	254	297	3,900
		(1,429 to 44,833)	(−2,416 to 22,637)	(−5,741 to 52,258)	(−7,145 to 30,559)	(−17,689 to 20,497)
	*p-*value	0.253	**0.003**	**0.004**	**0.004**	**0.004**
MIP-1α	Patients	19	284	269	123	2,107
		(1 to 505)	(17 to 5,688)	(0 to 1,000)	(18 to 2,690)	(−49 to 13,538)
	Controls	315	24	221	1	6**
		(6 to 5,950)	(0 to 846)	(−10 to 639)	(−2,153 to 61)	(−4,872 to 74)
	*p-*value	0.410	0.272	0.465	**0.028**	**0.047**
MIP-1β	Patients	289	13,458	15,682	18,155	25,109
		(154 to 15,734)	(3,933 to 29,494)	(3,702 to 29,263)	(2,105 to 29,484)	(4,745 to 37,410)
	Controls	336	111	59	106	−74
		(33 to 453)	(−4 to 272)	(−66 to 426)	(−324 to 590)	(−361 to 418)
	*p-*value	0.465	**0.006**	**0.006**	**0.006**	**0.009**
MMP-9	Patients	185,949	271	15,740	15,639	−148
		(94,883 to 326,513)	(28 to 98,216)	(−45 to 119,810)	(−35,972 to 121,997)	(−81,276 to 121,819)
	Controls	219,901	9,230	52,022	40,621	210**
		(94,886 to 379,856)	(−32,479 to 88,506)	(0 to 93,451)	(−28,509 to 144,182)	(−35,664 to 126,870)
	*p-*value	0.584	0.855	0.855	0.855	0.465
RANTES	Patients	20,988	548	5,149	−12	3,238
		(8,034 to 42,202)	(−29,902 to 74,284)	(−5,196 to 74,287)	(−33,996 to 74,252)	(−39,618 to 74,266)
	Controls	25,468	2	720	5,213	2,871
		(8,033 to 43,474)	(−12,265 to 7,702)	(−21,765 to 28,541)	(−29,131 to 36,499)	(−33,264 to 20,574)
	*p-*value	0.886	0.475	0.668	1.000	0.522
TNF-α	Patients	2	1	0	36	143
		(0 to 207)	(−42 to 77)	(−53 to 29)	(−69 to 127)	(−115 to 173)
	Controls	15	0	16	6	−1
		(1 to 4,590)	(−310 to 231)	(−842 to 721)	(−1,191 to 361)	(−1,656 to 94)
	*p-*value	0.223	0.871	0.806	0.570	0.100
VEGF	Patients	306*	−40*	−64*	−203*	−139*
		(0 to 400)	(−179 to 25)	(−191 to 248)	(−277 to 35)	(−216 to 0)
	Controls	335	−17	28	11	−4**
		(35 to 432)	(−114 to 90)	(−44 to 162)	(−61 to 164)	(−87 to 180)
	*p-*value	1.000	0.831	0.522	0.055	0.142

Median background cytokine concentrations (Nil, pg/mL) and cytokine responses (pg/mL) to phytohemagglutinin (Mitogen Response), early secreted antigenic target-6 (ESAT-6 Response), culture filtrate protein 10 (CFP-10 Response), TB7.7 (TB7.7 Response), and combinations of these *M. tuberculosis* (*Mtb*) antigens (ESAT-6+CFP-10 Response and ESAT-6+CFP-10+TB7.7 Response) are listed with ranges in parentheses. Cytokine concentrations were measured by a commercial quantitative immuno-microaray (microarray) for patients with culture-confirmed tuberculosis (patients) and subjects at low risk for *Mtb* exposure (controls). Responses were calculated by subtracting the background cytokine concentration in plasma from blood incubated with saline (Nil) from the cytokine concentration in plasma from blood incubated with mitogen or *Mtb* antigens. Cytokine responses for some patients and controls were not determined due to poor performance of the respective standard curves. N = 5 or 6 for patients subjects and N = 6 or 7 for controls unless indicated (*n = 4, **n = 5). *P* values were calculated by Mann-Whitney U Rank Sum test comparing cytokine concentrations or responses for patients with controls. Significant differences (*p*<0.05) are indicated in **bold type**.

**Table 8:** Comparisons of cytokine responses to different antigens measured by Microarray among patients with culture-confirmed tuberculosis.

Cytokine	ESAT-6	ESAT-6	ESAT-6	CFP-10	CFP-10	ESAT-6+CFP-10
	*vs.*	*vs.*	*vs.*	*vs.*	*vs.*	*vs.*
	CFP-10	ESAT-6+CFP-10	ESAT-6+CFP-10+TB7.7	ESAT-6+CFP-10	ESAT-6+CFP-10+TB7.7	ESAT-6+CFP-10+TB7.7
IFN-g	682	682	682	763	763	1,270
	vs.	vs.	vs.	vs.	vs.	vs.
	763	1,270	1,259	1,270	1,259	1,259
	(0.463)	(0.116)	(0.345)	(0.249)	(0.345)	(0.463)
IL-2	428	428	428	499	499	740
	vs.	vs.	vs.	vs.	vs.	vs.
	499	740	746	740	746	746
	(0.249)	**(0.046)**	**(0.046)**	(0.116)	(0.116)	(0.249)
IL-6	250	250	250	28	28	182
	vs.	vs.	vs.	vs.	vs.	vs.
	28	182	781	182	781	781
	**(0.046)**	(0.917)	(0.173)	(0.116)	**(0.028)**	(0.249)
IL-8	1,038	1,038	1,038	1,988	1,988	1,595
	vs.	vs.	vs.	vs.	vs.	vs.
	1,988	1,595	3,076	1,595	3,076	3,076
	(0.273)	(0.068)	(0.144)	(0.715)	(0.715)	(0.715)
MCP-1	72,572	72,572	72,572	102,845	102,845	76,846
	vs.	vs.	vs.	vs.	vs.	vs.
	102,845	76,846	72,438	76,846	72,438	72,438
	(0.600)	(0.686)	(0.345)	**(0.043)**	(0.917)	(0.893)
MIP-1α	284	284	284	269	269	123
	vs.	vs.	vs.	vs.	vs.	vs.
	269	123	2,107	123	2.107	2,107
	(0.686)	(0.686)	(0.138)	(0.345)	(0.138)	(0.138)
MIP-1β	13,458	13,458	13,458	15,682	15,682	18,155
	vs.	vs.	vs.	vs.	vs.	vs.
	15,682	18,155	25,109	18,155	25,109	25,109
	(0.893)	(0.893)	(0.686)	(0.893)	(0.138)	(0.686)

Median cytokine responses (from Tables 6 and 7) to early secreted antigenic target-6 (ESAT-6), culture filtrate protein 10 (CFP-10), and combinations of these *M. tuberculosis* (*Mtb*) antigens (with or without TB7.7) for patients with culture-confirmed tuberculosis (patients) are listed with p values in parenthesis calculated by Mann-Whitney U Rank Sum test. Significant differences (*p*<0.05) are indicated in **bold type**.

Additional samples were analyzed to confirm findings from the previous two assays. For this analysis a custom commercial MMIA was chosen and a chemokine not yet tested, IP-10, was included. As shown in Table 9, IFN-γ, IL-2, IL-6, IL-8, IP-10, MCP-1, MIP-1β and TNF-α responses were significantly greater for CCTB patients compared with controls after whole blood was stimulated with ESAT-6+CFP-10+TB7.7. In addition, background IP-10, MCP-1, and MIP-1β concentrations in plasma from unstimulated blood (Nil) from patients were significantly greater than from controls. IL-12 responses were low and not significant. Another observation was that the IL-2 Mitogen response was significantly greater for controls versus patients, which was also suggested by the results shown in Table 4, but was not significant.

**Table 9:** Cytokine concentrations and responses measured by a commercial MMIA.

Cytokine	Subject Group	Nil	Mitogen Response	ESAT-6+CFP-10+TB7.7 Response
IFN-γ	Patients	165	2,002	913
		(0 to 885)	(502 to 3,791)	(108 to 2,974)
	Controls	79	1,930	38
		(0 to 827)	(766 to 3,459)	(−8 to 190)
	*p-*value	0.184	0.908	**<0.001**
IL-2	Patients	10	332	286
		(0 to 35)	(30 to 1,081)	(37 to 1,553)
	Controls	6	590	2
		(0 to 63)	(160 to 1,340)	(−7 to 11)
	*p-*value	0.222	**0.033**	**<0.001**
IL-6	Patients	34	6,941	438
		(0 to 156)	(222 to 25,260)	(58 to 1,302)
	Controls	11	5,146	11
		(0 to 49)	(608 to 9,632)	(−2 to 46)
	*p-*value	0.202	0.817	**<0.001**
IL-8	Patients	199	17,810	2,682
		(40 to 819)	(227 to 90,979)	(135 to 21,942)
	Controls	247	9,182	170
		(41 to 635)	(2,093 to 29,080)	(−22 to 735)
	*p-*value	0.166	0.564	**<0.001**
IL-12	Patients	0	4	0
		(0 to 0)	(0 to 19)	(0 to 0)
	Controls	2	4	0
		(0 to 25)	(0 to 18)	(0 to 1)
	*p-*value	0.317	0.829	0.317
IP-10	Patients	3,807	26,252	39,065
		(143 to 25,641)	(6,786 to 58,752)	(2,530 to 158,425)
	Controls	589	25,663	21
		(149 to 1,275)	(5,687 to 62,714)	(−202 to 349)
	*p-*value	**0.033**	0.954	**<0.001**
MCP-1	Patients	2,879	20,372	13,729
		(39 to 12,764)	(4,842 to 44,202)	(−221 to 39,519)
	Controls	290	19,525	494
		(28 to 1,319)	(4,418 to 44,461)	(−72 to 1,899)
	*p-*value	**0.024**	0.908	**<0.001**
MIP-1β	Patients	373	47,076	8,245
		(60 to 1,389)	(20,657 to 75,448)	(773 to 24,204)
	Controls	180	46,596	64
		(59 to 415)	(20,537 to 75,511)	(0 to 198)
	*p-*value	**0.021**	0.817	**<0.001**
TNF-α	Patients	65	3,268	303
		(0 to 329)	(250 to 6,715)	(42 to 719)
	Controls	9	1,470	14
		(0 to 105)	(491 to 3,910)	(0 to 94)
	*p-*value	0.074	0.184	**<0.001**

Median background cytokine concentrations (Nil, pg/mL) and cytokine responses (pg/mL) to phytohemagglutinin (Mitogen Response) and to a combination of early secreted antigenic target-6, culture filtrate protein 10, and TB7.7 (ESAT-6+CFP-10+TB7.7 Response) are listed with ranges in parentheses. Cytokine concentrations were measured with a custom commercial multiplexed microsphere-based immunoassay (commercial MMIA) for patients with culture-confirmed tuberculosis (patients) and subjects at low risk for *M. tuberculosis* (*Mtb*) infection (controls). For values that were designated “out of range” by Bio-Plex Manager software, low values are reported as 0 and high values are reported as the observed value of the highest standard concentration multiplied by the dilution factor. Responses were calculated by subtracting the background cytokine concentration in plasma from blood incubated with saline (Nil) from the cytokine concentration in plasma from blood incubated with mitogen or *Mtb* antigen. N = 12 for patients and N = 12 for controls. *P* values were calculated by Mann-Whitney U Rank Sum test comparing cytokine concentrations or responses for patients versus controls. Significant differences (*p*<0.05) are indicated in **bold type**.

**Figure 2:**
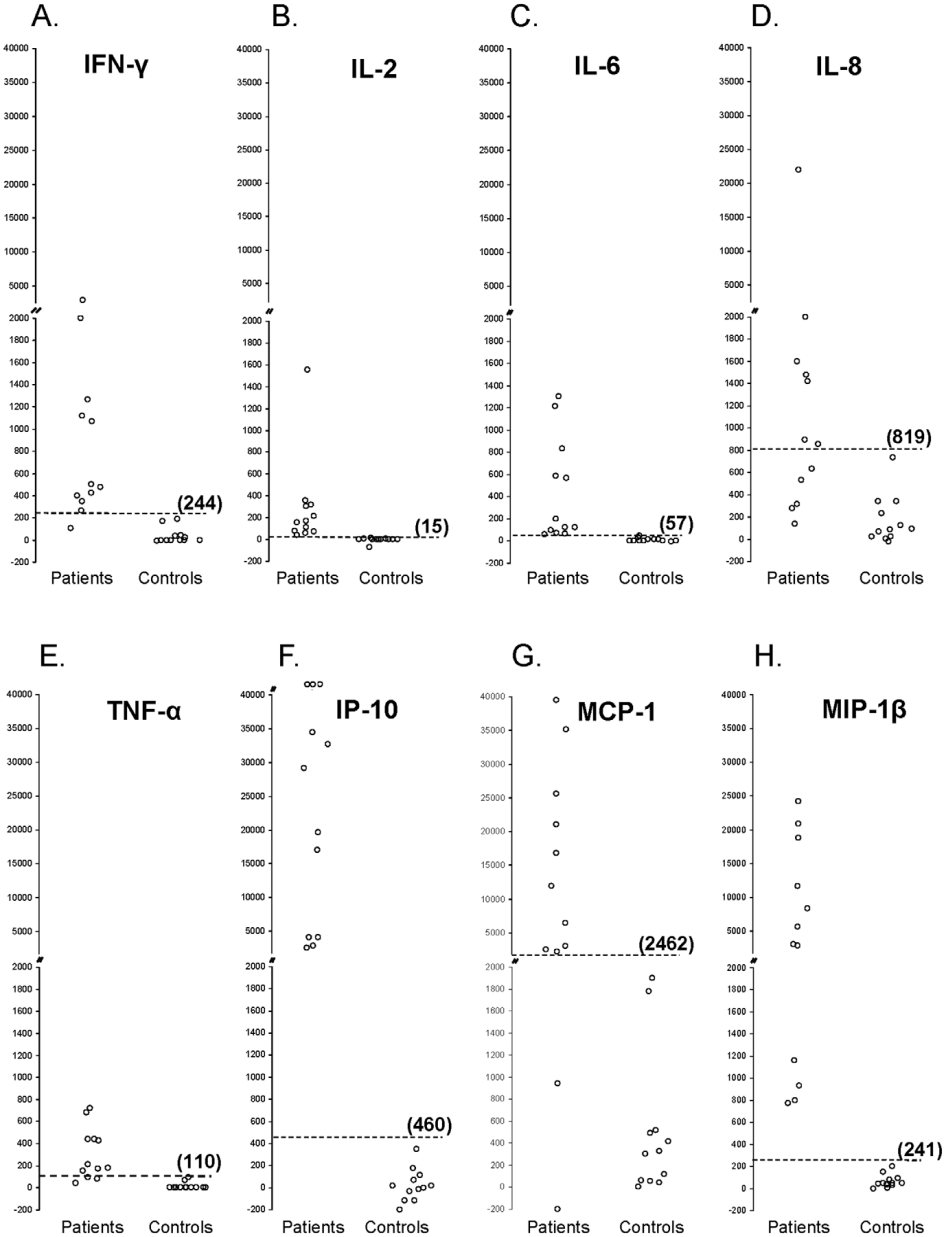
(A–H): Responses of eight cytokines to specific *M. tuberculosis* antigens. Cytokine responses to a peptide cocktail representing early secreted antigenic target-6, culture filtrate protein 10, and TB7.7 (ESAT-6+CFP-10+TB7.7) are depicted for the 12 patients with culture-confirmed tuberculosis (patients) and 12 subjects at low risk for *Mtb* infection (controls) represented in Table 9. Cytokine concentrations were measured with a custom commercial multiplexed microsphere-based immunoassay (commercial MMIA). Note the split y-axis. The dotted line and value in parentheses represents the mean cytokine response for controls plus three standard deviations, which was used as a cut point to separate positive and negative responses. **Panels A through H** depict IFN-γ, IL-2, IL-6, IL-8, TNF-α, IP-10, MCP-1, and MIP-1β responses.

**Figure 3:**
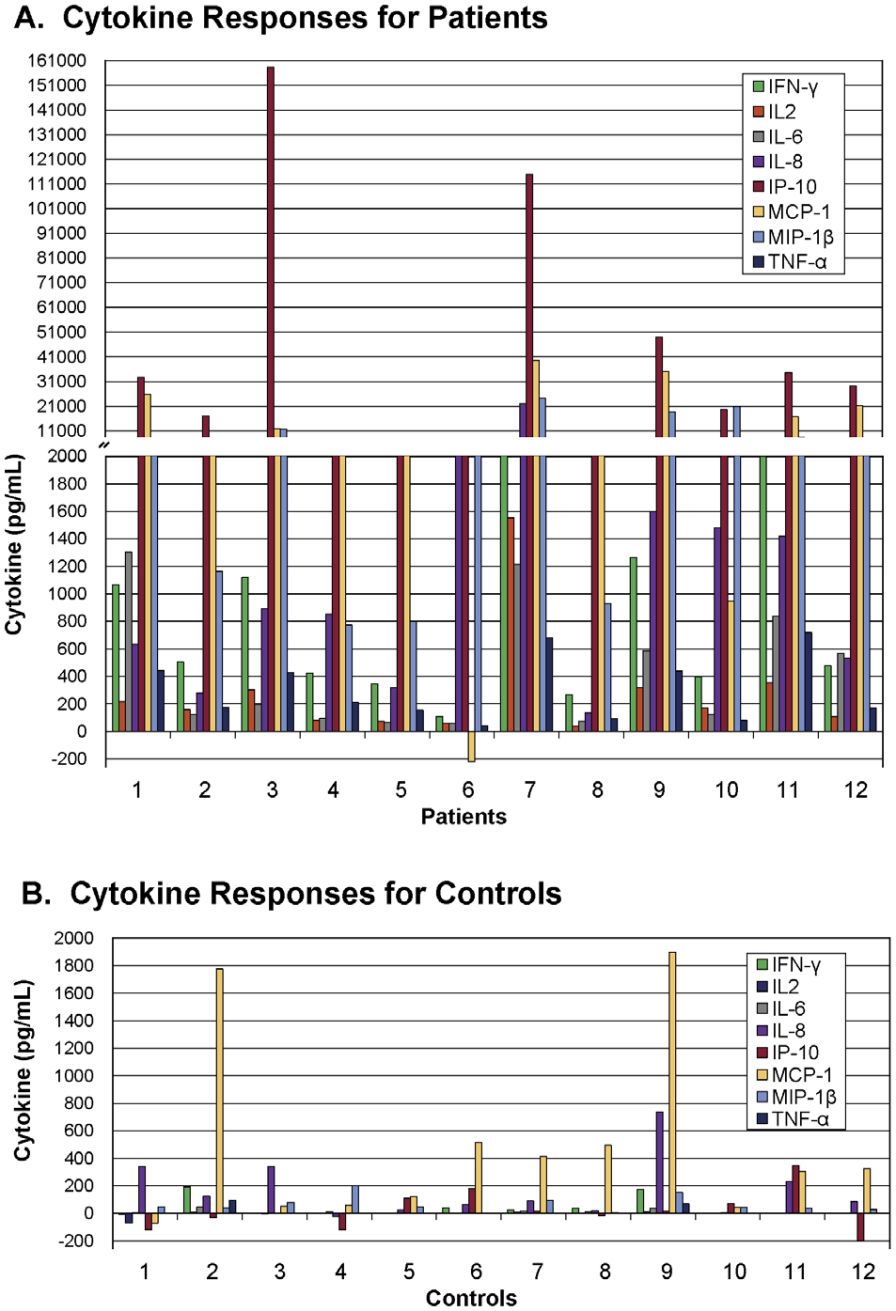
(A–B): Patterns of cytokine responses. Patterns of cytokine response to a peptide cocktail representing early secreted antigenic target-6, culture filtrate protein 10, and TB7.7 (ESAT-6+CFP-10+TB7.7) are depicted for 12 patients with culture-confirmed tuberculosis (patients) in **Panel A,** and for 12 subjects at low risk for *Mtb* infection (controls) in **Panel B** that were summarized in Table 9. Cytokine concentrations were measured with a custom commercial multiplexed microsphere-based immunoassay (commercial MMIA). Responses were calculated by subtracting the background cytokine concentration in plasma from blood incubated with saline (Nil) from the cytokine concentration in plasma from blood incubated with *Mtb* antigens.

Dot-plots (Figures 2A–H) and histograms (Figures 3A–B) showing patterns of cytokine responses represented in Table 9, illustrate the dramatic cytokine activity that occurs when whole blood from CCTB patients are stimulated with ESAT-6+CFP-10+TB7.7. The split y-axis at 2000 pg/mL highlights the tremendous IP-10, MCP-1, and MIP-1β responses seen for the majority of patients. Sensitivity and specificity were 100% with IL-2, IL-6, IP-10, and MIP-1β, based on cutoffs equal to the mean responses to ESAT-6+CFP-10+TB7.7 plus three standard deviations for control subjects. With IFN-γ measured by the custom commercial MMIA, similar calculations demonstrated a specificity of 100% with a sensitivity of 91.6%. One CCTB patient classified as negative based on the IFN-γ response was considered positive based on the responses of IL-2, IL-6, IL-8, IP-10, or MIP-1β. This subject was also classified as negative based on the IFN-γ response to ESAT-6+CFP-10+TB7.7 as well as other *Mtb* antigens measured by commercial ELISA.

## Discussion

The sensitivity of FDA approved IGRAs is less than ideal, and typically no better than the sensitivity of the TST [3], [4]. This pilot study was designed to investigate the use of other cytokines as markers of *Mtb* infection and to evaluate the potential impact of their use on detection sensitivity. Cytokine responses to individual *Mtb* antigens and antigen combinations were compared.

Each of three multiplexed assays demonstrated that in addition to IFN-γ, multiple cytokines are released in response to *Mtb* antigens with significantly greater responses among patients with CCTB than among control subjects. The in-house MMIA and microarray demonstrated that IFN-γ, IL-2, IL-6, and IL-8 responses to the combination antigen ESAT-6+CFP-10+TB7.7 were greater among patients than among controls. In addition, the microarray data demonstrated significantly greater MCP-1, MIP-1α, and MIP -1β responses among patients. The custom commercial MMIA confirmed that multiple cytokines were released in response to *Mtb* antigens, and that IFN-γ, IL-2, IL-6, IL-8, IP-10, MCP-1, MIP-1β, and TNF-α responses to ESAT-6+CFP-10+TB7.7 were significantly greater for patients than for controls. Four of these cytokines appear to be excellent candidates as diagnostic markers of *Mtb* infection; sensitivity and specificity were 100% with IL-2, IL-6, IP-10, and MIP-1β. In contrast, IFN-γ sensitivity with the custom commercial MMIA was 91.6% and specificity was 100%.

One CCTB patient failed to demonstrate an IFN-γ response with any of the *Mtb* antigens using multiple test formats including QFT-G. The subject had a robust IFN-γ response to mitogen, no other evidence of immune dysfunction, nor any unusual IFN-γ measurements to suggest laboratory errors. In stark contrast to the lack of IFN-γ response, this subject's IL-2, IL-6, IL-8, IP-10, and MIP-1β responses to ESAT-6+CFP-10+TB7.7 were greater than corresponding mean responses plus three standard deviations for controls. This observation demonstrates that the use of other cytokines in addition to IFN-γ or as alternatives to IFN-γ can improve sensitivity for detecting *Mtb* infection.

Plausible reasons for false-negative IGRA results include: 1) measurement errors [34], 2) host-mediated inhibition of IFN-γ release [35], 3) drug or disease induced immune suppression [36], [37], 4) genetic defects in IFN-γ production [38], [39], and 5) Major Histocompatibility Complex (MHC) determined restriction in the ability to recognize presented epitopes [40]. The potential for improving diagnostic sensitivity by assessing responses of multiple cytokines has been suggested previously [23]. By assessing both IP-10 response and IFN-γ response, Ruhwald et al. noted an increase in detection rate to 90% (i.e., detection by either cytokine) from 83% and 81%, respectively, for IP-10 or IFN-γ alone. Individual cytokines have been found to be down-regulated in some patients with tuberculosis, suggesting that assessment of multiple cytokines may reduce the possibility of a negative response with any single analyte [41].

The amount of cytokine in unstimulated plasma from patients with CCTB was generally not significantly different from the amount in unstimulated plasma from control subjects. However, background levels of IP-10, MCP-1, and MIP-1β measured with the custom MMIA were statistically higher for patients than for controls. This observation has also been made for IP-10 and MCP-3 previously [24], [42]. The specificity and sensitivity of this observation for *Mtb* infection should be further investigated. If these cytokines are consistently elevated without antigen stimulation in *Mtb* infected persons, as opposed to those with other infections, they could serve as the basis for a more rapid test for *Mtb* infection. If antigen stimulation is not required, detection of elevated cytokine levels in the urine may be of diagnostic value [43].

IP-10 has been targeted as a potential diagnostic marker for tuberculosis in other studies [20]–[24], [26], [42], [44]–[48]. In conjunction with our results, these studies provide clear evidence that IP-10 is produced in nanogram amounts when fresh blood samples from most sensitized persons are stimulated with mycobacterial antigens. Azzurri et al. noted an association between unstimulated IP-10 plasma levels and disease activity, as well as response to treatment [20]. In a pilot study, Syed Ahamed Kabeer et al. found that tuberculosis treatment was associated with a significant decrease in IP-10 response to certain peptides representing ESAT-6 and CFP-10 [49]. Other promising indicators of *Mtb* infection include antigen-induced MCP-2, MCP-3, and IL-1RA responses [23], [24], [42], [44].

The antigen or combination of antigens used to stimulate blood can have a dramatic impact on the magnitude of the cytokine response observed. Among sensitized persons, the number of epitopes in an antigen and Major Histocompatibility Complex (MHC) restrictions determines the number of T-lymphocyte clones that will be stimulated by the antigen, and in turn, affects the amount of IFN-γ released upon stimulation [40], [50]. For example, among CCTB patients, IFN-γ responses measured by ELISA to ESAT-6+CFP-10 or ESAT-6+CFP-10+TB7.7 were greater than the response to any of the individual antigens alone. However, the IFN-γ response to ESAT-6+CFP-10+TB7.7 was not significantly greater than the response to ESAT-6+CFP-10. This trend toward higher response to the combination antigens continued for other cytokines measured with the in-house MMIA. For this reason, and because ESAT-6+CFP-10+TB7.7 is used in the QuantiFERON®-TB Gold In-Tube test, only responses to ESAT-6+CFP-10+TB7.7 were measured with the custom commercial MMIA.

Cytokine levels vary considerably from individual to individual, so the range of values observed with the multiplexed assays was rather large. Despite these large variations and the relatively small sample sizes, significant differences between CCTB patients and controls were observed, and measurements of IL-2, IL-6, IP-10, and MIP-1β responses allowed complete separation of the two groups. The dot plots and histograms (Figures 2 and 3) illustrate the multiple sizeable cytokine responses and the pattern seen among patients. For IP-10 and MIP-1β, the magnitude of the responses to *Mtb* antigens was so large compared to background cytokine levels that subtraction of the background had little effect on the calculated responses. This has two implications. First, the complexity and cost of testing may be reduced by screening only *Mtb*-antigen stimulated plasma for characteristically high cytokine concentrations. If increased concentrations of IP-10 or MIP-1β are found in the Mtb-antigen stimulated plasma, a more sensitive and complex assay could be performed that would take into account the background cytokine concentrations. Second, significant cytokine responses may be detectable with shorter incubation times, thus allowing for more rapid assessments.

Differences are often seen in the magnitude of cytokine concentrations measured with different ELISAs or different multiplexed immunoassay systems [51]. These differences are primarily due to varying antibody affinities for the assay targets, as well as differences in the platforms employed. We used three multiplexed immunoassay systems that assessed some of the same cytokines with presumably different capture and detection antibodies. In all three systems, differences in IL-2, IL-6, IL-8, and IFN-γ responses between CCTB patients and controls were significant. In two systems, differences in MCP-1 and MIP-1β responses were significant. TNF-α responses were significantly greater among patients versus controls using the in-house MMIA and commercial MMIA, but not when using the microarray. However, TNF-α concentrations were generally low and highly variable, which may explain why the differences in TNF-α response were not significant for all three assays.

Limitations of this study stem from the examination of small subsets of subjects with each of the different multiplexed immunoassays. However, even with small numbers of subjects significant differences in cytokine responses were demonstrated between CCTB patients and controls. While differences in demographic and clinical characteristics for the patient and control populations were observed (listed in Table 1), it is unlikely that these explain the differences in cytokine responses seen. Subtracting the background cytokine concentration (in the Nil plasma) from the concentration in the antigen stimulated plasma adjusts for demographic and clinical factors that may contribute to nonspecific release of cytokines. The observed differences in unstimulated concentrations for some cytokines (including IFN-γ) is likely due to elevated background levels of cytokine associated with acute or chronic illness, including tuberculosis. Another limitation is the lack of adequate diagnostic standards to confirm all forms of *Mtb* infection, including the most common, which is latent infection. We addressed this limitation by comparing responses among patients with confirmed *Mtb* infection (i.e., among patients with tuberculosis confirmed by recovery of *Mtb* by culture) and control subjects at low-risk of *Mtb* exposure who were presumed to be uninfected. Clearly, immunologic differences exist between latent *Mtb* infection and infection manifesting as active disease, but specific immunologic differences have been difficult to demonstrate [26], [52]. Larger epidemiologically based studies will be required to identify and confirm these differences. Multiplexed assays, which can be readily automated for high-throughput, can facilitate these larger studies.

MMIAs, which are also referred to as suspension arrays, are gaining wide acceptance as tools for research and diagnostic assays [53], [54]. These assays are performed in 2 to 4 hours, similar to the time required to perform ELISAs. However, in addition to measuring more than one analyte per test, MMIAs require smaller sample volumes (4 µL in this study), less amounts of reagent and operator time, and cost less than multiple ELISAs. These three dimensional assays have a detection range 1–2 logs broader than ELISAs, so fewer sample dilutions are necessary. Already a simpler, less expensive instrument is available that is not dependent on the fluidics system of a flow cytometer (MAGPIX®, Luminex Corporation). This and similar systems are more compact and portable and show potential for use in developing regions of the world where both reduction in assay cost and time delay between diagnosis and treatment are important. As multiplexed assays that show potential for detection of *Mtb* infection are optimized, they can be incorporated onto other platforms that are fully automated or designed for point-of-care diagnostics.

In conclusion, multiple cytokines are released when whole blood samples from patients with CCTB are stimulated with specific *Mtb* antigens. Compared with measurement of IFN-γ alone, multiplexed assays can improve diagnostic sensitivity for *Mtb* infection by assessing multiple cytokine responses simultaneously, using smaller sample volumes and reducing the cost of testing compared to ELISAs and other methods. The use of combinations of specific *Mtb* antigens may also increase diagnostic sensitivity by increasing the magnitude of cytokine responses as compared to individual antigens. Larger studies are needed to confirm the clinical utility of multiplexed assays that examine the cytokines identified in this study as potential diagnostic markers of *Mtb* infection and to examine the possibility of differentiating infection with disease from latent infection.
